#  A Modified Stoppa (Technique) Approach for Treatment of Pediatric Acetabular Fractures

**DOI:** 10.1155/2013/478131

**Published:** 2013-05-27

**Authors:** Mehmet Elmadag, Mehmet Ali Acar

**Affiliations:** ^1^Department of Orthopedics and Traumatology, Medical Faculty, Bezmialem Vakif University, Fatih, 34093 Istanbul, Turkey; ^2^Department of Orthopedics and Traumatology, Selcuklu Medicine Faculty, Selcuk University, Konya, Turkey

## Abstract

Pediatric acetabular fractures are rare, and anterior column fractures are even rarer. Generally, conservative treatment is applied. If there is displacement of more than 2 mm or findings of instability or fragments within the joint, then surgical treatment is applied. Anterior and posterior approaches may be used in surgical treatment. With pediatric patients, even greater care should be taken in the choice of surgery to be performed according to the fracture pattern to avoid postoperative triradiate cartilage damage. Therefore, minimally invasive surgery is more appropriate. We herein present a case of an acetabulum anterior column posterior hemitransverse fracture following a traffic accident, which was treated surgically using a modified Stoppa (technique) approach.

## 1. Introduction

Acetabular fractures are rare in childhood [1, 2] because increased cartilage volume, joint elasticity, and strong ligaments in children allow for significantly higher energy absorption prior to fracture [3]. Therefore, the formation of a fracture pattern that will require surgery is not often seen. Because of this rarity, nonsurgical treatment has been applied to fractures that require surgery. However, several studies have reported the results of this treatment to be unsatisfactory [4–7].

Open reduction internal fixation is recommended if there is displacement of the articular surface of more than 1 to 2 mm [8–10]. Surgical treatment is planned according to the area in which the displacement is greater. In cases with anterior displacement, a standard ilioinguinal approach is used. To minimize the potential complications associated with aggressive surgery in children, minimally invasive surgery is more appropriate.

The aim of this paper is to present a modified Stoppa approach with an open reduction and fixation technique for the surgical treatment of an acetabulum anterior column fracture, which to the best of our knowledge has not been previously reported in pediatric patients. 

## 2. Case Report

**Figure 1:**
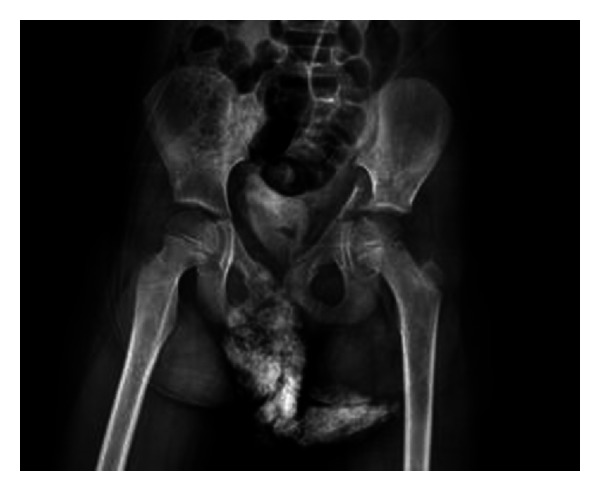
Preoperative pelvis AP X-ray.

**Figure 2:**
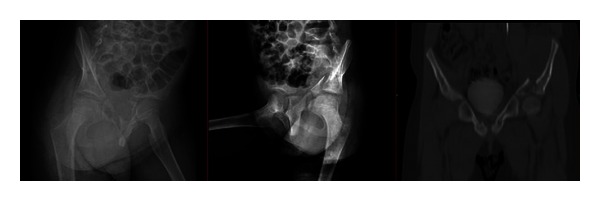
Preoperative iliac oblique and obturator oblique X-ray and pelvic CT.

A 7-year-old female patient who had been involved in a traffic accident while seating in the rear of a vehicle was transferred from an external hospital. On presentation, the patient reported left lower extremity numbness, pain, restricted joint movement, and pain and limited movement of the left arm. The patient had no neurovascular pathology. The initial radiographs showed a displaced fracture of the left acetabulum and a proximal fracture of the left humerus (Figure 1). Additional tests ruled out any other organ injury. Iliac oblique and obturator oblique X-rays and pelvic CT images were taken (Figure 2). Because these images demonstrated a displaced acetabulum anterior column posterior hemitransverse fracture, the patient was admitted for surgery.

**Figure 3:**
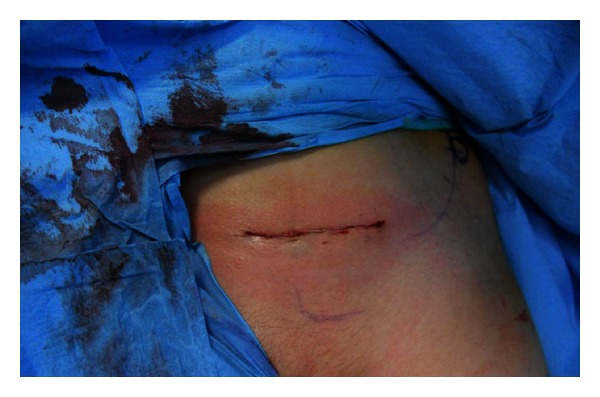
Clinical view of Pfannenstiel incision.

**Figure 4:**
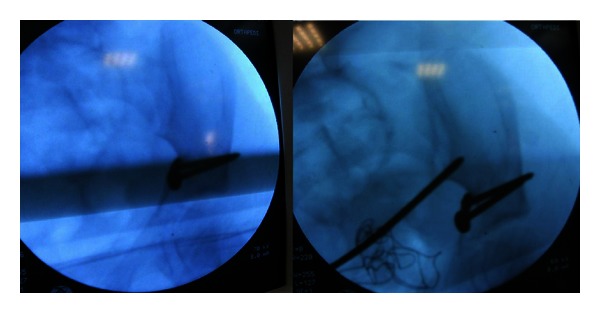
Use of the ball-spike pusher under fluoroscopy.

**Figure 5:**
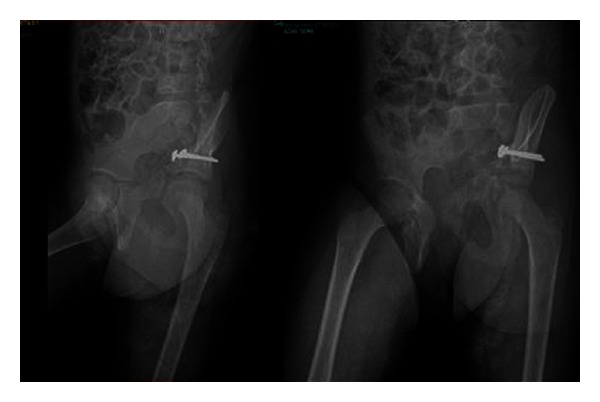
Early postoperative pelvis AP X-ray.

The patient was placed in a supine position on a radiolucent table. An intraoperative examination was performed under general anesthesia. Surgery was performed when it was established that closed reduction could not be applied to the fracture fragments. Because a modified Stoppa (technique) approach was suitable, the linea alba was opened longitudinally with a Pfannenstiel incision (Figure 3). A retractor was placed to protect the bladder, and the fracture fragments were reached from beneath the iliopsoas muscle. Fracture reduction was performed with the help of a ball-spike push (Figure 4). The reduction was maintained with two 3.5 mm screws placed using the lag fixation technique (Figure 5). Fluoroscopic guidance was used to ensure that the fixation avoided the triradiate cartilage. Closed reduction was then applied to the proximal humerus fracture, and fixation was completed with three K-wires. To allow evaluation of the postoperative joint movement and fracture stability, no spica cast was applied. The patient was hemodynamically stable during the first postoperative 24 h of monitoring and was discharged on postoperative day 2.

The sutures were removed at the second week of followup. The patient could not be mobilized because of the proximal humerus fracture; thus, partial weight-bearing mobilization was allowed in the sixth postoperative week.

**Figure 6:**
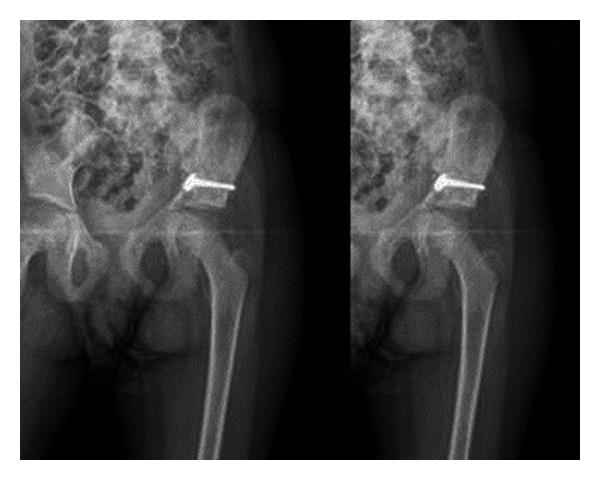
Twelve-month postoperative pelvis AP X-ray showing no cartilage damage.

The patient made good subjective progress, with no pain in her left hip or left humerus. At postoperative week 8, she was able to walk without support. After a 1-year follow-up period, her d'Aubigne and Postel [11] score was excellent (6-6-6) (Figure 6). The patient was able to walk and run without pain, had equal leg lengths, and had a full range of motion in the left hip. 

## 3. Discussion 

Acetabular fractures are diagnosed with an annual incidence of about 1 per 100,000 children [12]. Posterior acetabular wall fractures are the most common acetabular injuries, followed by transverse fractures with triradiate cartilage damage [1]. Anterior column injuries are seen much less frequently.

There is a scarcity of relevant cases in the literature, so no consensus on the ideal method of treatment has been reached. The majority of cases reported in the literature were treated nonsurgically [3]. Conservative treatment is indicated in simple nondisplaced fractures or those through nonweight-bearing areas [1]. This treatment comprises bed rest and skeletal traction for 4 to 8 weeks. On discontinuing traction, toe-touch weight bearing is initiated and slowly advanced to full weight bearing over the subsequent 6 weeks [3]. However, multiple case series and retrospective studies have shown that nonsurgical treatment of displaced acetabular, sacroiliac, and vertically unstable pelvic fractures is associated with residual low-back pain, pelvic asymmetry, and leg-length discrepancy [4–7]. Open reduction internal fixation is recommended if there is displacement of the articular surface of more than 1 to 2 mm [13]. The quality of reduction has been correlated with the clinical results of acetabular fractures [8]. However, the relationship between the clinical outcome and displacement has not been described as clearly in children [2]. 

Together with surgical treatment, early mobilization and early physical therapy are started. The approach selected for surgical treatment depends on the fracture pattern. The Kocher-Langenbeck approach is preferred for the frequently seen posterior wall fracture pattern [14]. Although anterior injuries are encountered less commonly, the approach reported in the literature is ilioinguinal approach of Letournel and Judet [15]. In the case presented herein, a modified Stoppa (technique) approach with anterior column fracture fixation was used.

The midline approach for hernia treatment with a complicated incision was first described by Stoppa in 1989. In 1994, Cole and Bolhofner began to use it successfully as minimally invasive acetabulum surgery in adult patients [16]. Recent studies have reported fewer complications and less morbidity from the Stoppa approach [17, 18]. Because the case presented here involved a 7-year-old female patient, open reduction and internal fixation using the Stoppa approach were as minimally invasive as possible. 

The most significant complication seen in these types of injuries is triradiate cartilage damage. The most important factor indicating the prognosis is the patient's age at the time of the accident. If the patient is younger than 10 years, the risk of damage is high [1, 3, 13]. Triradiate cartilage damage may be associated with surgery and not with the initial trauma. Therefore, the treatment for these types of fractures in pediatric patients should be minimally invasive. In the case presented here, modified Stoppa surgery was the preferred minimally invasive surgical technique, and after a 1-year followup, no cartilage damage was found.

## 4. Conclusion

Anterior acetabulum fracture surgery is extremely rare in pediatric patients, and an ilioinguinal approach is generally used. The results of the case presented here demonstrate that the modified Stoppa (technique) approach, which has been used successfully in adult patients, can also be used in pediatric patients.
